# Acute impact of a national lockdown during the COVID-19 pandemic on wellbeing outcomes among individuals with chronic pain

**DOI:** 10.1177/1359105321995962

**Published:** 2021-02-18

**Authors:** Zoë Zambelli, António R Fidalgo, Elizabeth J Halstead, Dagmara Dimitriou

**Affiliations:** 1 University College London-Institute of Education, UK; 2 University of East London, UK

**Keywords:** anxiety, chronic pain, Covid-19, depression, sleep

## Abstract

Changes to wellbeing in a community-based sample of 638 adults with non-malignant chronic pain were assessed during a period of mandated lockdown measures in the UK to control the COVID-19 outbreak. Participants completed an online survey pre-lockdown and were followed up during lockdown. Multivariate analysis demonstrated that decreased ability to self-manage pain, restricted access to healthcare and increased dependence on others were associated with negative wellbeing outcomes related to sleep, anxiety and depression. Essential but non-urgent services are required during periods of lockdown to maintain independence and self-management in order to preserve wellbeing in this population.

## Introduction

Throughout 2020, the COVID-19 pandemic prompted governing bodies across the globe to impose combative public health measures to contain the virus and reduce infection rates and mortality (Chakraborty and Maity, 2020). In the United Kingdom (UK), measures included social distancing, closure of schools and public spaces, and a ‘stay at home’ order except in essential circumstances (Jarvis et al., 2020). These restrictions, described as ‘lockdown’, brought considerable disruption to social and economic activities, and reduced access to many essential but non-urgent services (Gupta et al., 2020; Iacobucci, 2020). Research during the pandemic has found evidence of high psychological distress in the general population across different cultures and regions (Liang et al., 2020; Odriozola-González et al., 2020; Xiong et al., 2020). Reasons for this may stem from a combination of factors which initiate and perpetuate psychological distress such as: health-related fear, isolation, confinement, misinformation, changes in daily routine and economic uncertainty (Giordani et al., 2020; Odriozola-González et al., 2020; Ornell et al., 2020).

### Impact on wellbeing among chronic pain populations

It is estimated that around one third of the UK population lives with a form of chronic pain and one in four individuals experience poor mental health at any one time (Fayaz et al., 2016b). The high prevalence of chronic pain and poor mental health negatively impacts individuals in terms of their wellbeing, and accounts for large losses of economic productivity at societal level. The biopsychosocial model of health implicates an interaction between biological, psychological, and social factors which affect an individual’s illness perception, pain-management and overall wellbeing (Bevers et al., 2016). Chronic pain populations report higher levels of anxiety, depression, post-traumatic stress disorder and poor sleep quality than the general population, as well as higher comorbidity of physical illnesses (Fayaz et al., 2016a; Gatchel, 2004; Morin et al., 1998). The relationship between sleep and pain has been shown to be bidirectional and particularly impacts this population. Pain may impact sleep by increasing micro-arousals, which over time cause fragmented sleep patterns. Equally, sleep disturbance has been shown to reduce pain thresholds with severe consequences for chronic pain populations who experience pain in episodic or persistent manners (Smith and Haythornthwaite, 2004).

Furthermore, chronic pain populations may be disproportionately impacted by public health restrictions during a pandemic because of reduced access to healthcare, risk of increased disease severity from contracting COVID-19, and closure of social and community support services (Kang et al., 2020; Rhodes et al., 2020). There is clear evidence that self-management strategies play an important role for psychological coping and management of chronic pain (Nicholas and Blyth, 2016). These strategies include adhering to prescribed medication or physical activity regimen, identifying treatments jointly with a health practitioner, in addition to managing the impact on mood and relationships due to pain interference. The ability to adopt self-management strategies has been shown to improve mental health in this population, however, evidence also suggests that socioeconomic status (SES) may influence self-management outcomes as those with low SES may have access to fewer resources than those with high SES (Hardman et al., 2020). Additionally, access to healthcare greatly impacts an individual’s health journey as those with chronic pain rely on a combination of assessments, diagnostics, and interventions, involving frequent interaction with the health system (Reid et al., 2015). Losing the ability to self-manage or restricting healthcare access for prolonged periods may have a significant impact on wellbeing including sleep behaviours and mental health. The pandemic brings risk and burden to chronic pain populations regarding disease management, as well as the potential to impact social and health behaviours. It is therefore important to study this population during periods of lockdown to ensure their wellbeing is not severely affected and mitigate risk in future waves where possible (Arora and Grey, 2020).

The objective of this study was to explore changes in wellbeing outcomes as related to sleep, anxiety and depression within a community sample of adults living with chronic pain between the start of the COVID-19 outbreak, pre-lockdown and during a period of lockdown in the UK. Two aims were identified to pursue the study:

(1) To measure changes in wellbeing outcomes (sleep, anxiety and depression) observed between the pre-lockdown period (Time 1) and during lockdown (Time 2).
(2) To identify if COVID-19 specific changes related to (a) dependence on others for support, (b) ability to self-manage pain condition and (c) access to healthcare were associated with wellbeing outcomes.

Given current global and national estimates of COVID-19, it is likely physical distancing measures and reduced service provision will continue for some time. Identifying factors which could impact wellbeing in this population may help health and social care services dealing with the pandemic’s response and recovery process.

## Methods

### Design

A cross-sectional survey was conducted related to a separate protocol pre-lockdown (Time 1: February and March 2020). To evaluate the impact of COVID-19 and the lockdown measures, a subsequent follow-up survey (Time 2: April and May 2020) was released to the same participants whilst lockdown measures were in place. Both surveys were hosted on Qualtrics (www.qualtrics.com). This research was granted ethical approval by UCL Institute of Education’s Ethics Committee. All participants were asked for explicit and voluntary consent at the start of each survey.

### Participants

A total of 1,234 adults with existing non-malignant chronic pain, who completed a cross-sectional survey at Time 1, were contacted to complete a follow-up study via online means at Time 2. A sample of 638 participants, living in the UK during the pandemic, responded and were included in this study (a response rate of 52%). During the analysis stage, participants with missing values for independent or dependent variables were excluded from individual analyses.

Participant characteristics are described in Table 1. Eligibility was based on participants being of adult age (18+ years), with an existing chronic pain condition unrelated to cancer. Participants were screened for mental and physical health conditions co-existing with their chronic pain condition. The sample was recruited via social media advertisements as well as online news bulletins and chronic pain charity websites. Participants did not receive compensation for taking part in the study.

**Table 1.** Participant characteristics.

Variable	Category	*N*	%	Mean + SD
Age		637		42.9 (13.4)
Gender	Male	70	11	
	Female	557	87	
	Prefer not to say	11	2	
Ethnicity	White – any background	601	94	
	Black/Black British	5	1	
	Asian/Asian British	7	1	
	Mixed	17	3	
	Other	6	1	
Highest level of education	Secondary school	70	11	
	Higher secondary or further Ed	185	29	
	Undergraduate degree	221	35	
	Postgraduate degree	154	25	
Cause of chronic pain	Chronic widespread pain	218	34	
	Musculoskeletal	234	37	
	Headaches	66	10	
	Neuropathic	93	15	
	Visceral	19	3	
	Other	8	1	
Time since pain inception	Up to 1 year	15	2	
	1–2 years	32	5	
	2–3 years	26	4	
	3–5 years	58	9	
	5–10 years	138	22	
	Over 10 years	365	57	
	Unsure	4	1	
Pain medication	Yes	574	90	
	No	64	10	
Co-morbid physical health condition^a^	Yes	605	95	
	No	33	5	
Co-morbid mental health condition^b^	Yes	353	55	
	No	285	45	

a Co-morbid physical health conditions such as diabetes, cardiovascular and respiratory conditions. ^b^Co-morbid mental health conditions such as depression and anxiety disorders.

### Measures

#### Background measures and independent variables related to the pandemic

At Time 1, background measures collected demographic indicators: age, sex, ethnicity, education status and a brief history in relation to participants’ chronic pain condition.

At Time 2, participants were asked questions relating to COVID-19 and personal circumstances during the lockdown period. These were converted into categorical variables and were used as independent variables during the analyses. Significant independent variables related to the COVID-19 lockdown were:

1. *‘Has your dependence on others such as family/friends/local community changed during the COVID-19 pandemic for practical and emotional support?’*
2. *‘Since the lockdown measures were put into place, have you felt any change in your ability to self-manage your pain?’*
3. *‘Has your access to healthcare (for non-COVID-19 related care) been affected by the COVID-19 pandemic?’*

Wellbeing measures related to anxiety, depression and sleep were collected during Time 1 and Time 2.

#### Wellbeing measure: Anxiety and depression

The Hospital Anxiety and Depression Scale (HADS) is a 14-item validated measure designed to measure anxiety and depression symptoms during the past week (Zigmond and Snaith, 1983). It comprises of two subscales assessing anxiety (HADS–A) and depression (HADS–D). Items are rated on a four-point Likert scale (e.g. 0 = *not at all* to 3 = *most of time*). Five items require reverse scoring. Scores for each item are summed for each subscale, scores above eight suggest anxiety and depression (Zigmond and Snaith, 1994). Cronbach’s alpha coefficients were found to be 0.83 for the anxiety subscale and 0.82 for the depression subscale (Bjelland et al., 2002).

#### Wellbeing measure: Sleep quality

The Pittsburgh Sleep Quality Index (PSQI) is a validated measure for sleep research and consists of 24 items (Buysse, 1989). The scale comprises seven components which measure (1) subjective sleep quality, (2) sleep latency, (3) sleep duration, (4) sleep efficiency, (5) sleep disturbance, (6) daytime dysfunction and (7) sleep medication over the past month. Each component generates a score from 0–3 where higher scores indicate poorer sleep outcomes. A sum of the seven component scores can be used to generate a global PSQI score ranging from 0 to 21. A global score above five indicates poor sleep quality. The scale has good reliability with Cronbach’s alpha scores above 0.8 (Carpenter and Andrykowski, 1998).

#### Pain

The Brief Pain Inventory (BPI) short form is a widely used self-report measure for clinical pain (Cleeland and Ryan, 1994). The BPI is composed of two subscales which rate severity of pain and the degree to which pain interferes with common dimensions of feeling and function in the past 24 hours. These are referred as (1) pain severity and (2) pain interference. Both subscales range from 0 to 10 with higher scores indicating higher levels of pain severity and interference. These scales have good internal consistency with Cronbach’s alpha of 0.85 and 0.88 for the severity and interference scales respectively (Tan et al., 2004).

#### Statistical analyses

To address the first aim and measure changes in wellbeing (sleep, anxiety and depression) and pain outcomes, paired *t*-tests were conducted to compare mean scores between Time 1 and Time 2 survey data.

To address the second aim and analyse the relationships between the grouping variables at Time 2; namely levels of dependence on others during lockdown, levels of ability to self-manage pain during lockdown, and access to healthcare during lockdown, and the dependent variables (global sleep quality, anxiety, and depression), three multivariate analysis of variance (MANOVA) were conducted. SPSS version 25 was used to conduct all analyses and significance levels were set at *p* ≤ 0.05.

#### Data sharing statement

The current article includes the complete raw dataset collected in the study including the participants’ data set, syntax file and log files for analysis. Pending acceptance for publication, all of the data files will be automatically uploaded to the Figshare repository.

## Results

Participants were mostly female (87%), of a white ethnic background (94%) and had a mean age of 42 years (SD = 13 years). Respondents were asked their chronic pain condition and responses were later categorised using the International Classification of Diseases (ICD) 11th edition which groups chronic pain into seven types (Treede et al., 2015). The sample included chronic widespread pain (e.g. fibromyalgia; 34%), musculoskeletal (e.g. osteoarthritis; 37%), headache (e.g. chronic migraine; 10%), visceral (e.g. pelvic pain; 3%), neuropathic (e.g. trigeminal neuralgia; 15%) and other (1%). Nearly all participants reported co-existing physical health conditions (95%), and over half reported a mental health condition (55%).

### Addressing Aim 1: Changes in wellbeing and pain outcomes

Table 2 displays the paired *t-*test results comparing mean scores across Time 1 (Feb-Mar) and Time 2 (Apr-May) for all eight PSQI components of sleep (global sleep quality, subjective sleep quality, sleep latency, sleep duration, sleep efficiency, sleep disturbance, daytime dysfunction and sleep medication), anxiety, depression, pain interference and pain severity. Across the whole sample, statistically significant improvements were reported at Time 2 in global sleep quality, subjective sleep quality, sleep duration, daytime dysfunction, depression, and pain outcomes compared to Time 1. These improvements did not reach the minimum importance difference (MID) of 1.75–3 points for global sleep quality, 1.7 points for depression or 2.2 points for pain severity (Lu et al., 2013; Mease et al., 2011; Puhan et al., 2008). There were no significant changes in anxiety levels between Time 1 and Time 2.

**Table 2.** Paired *t*-test results comparing averages for sleep, anxiety, depression, pain interference and pain severity at Time 1 and Time 2.

Variable	Time point	*N*	Mean + SD	*t*	*p* Value
Global sleep quality (PSQI)	Pre-lockdown	635	13.83 + 3.67	3.344	**0.001**
	During-lockdown		13.19 + 3.74		
Subjective sleep quality (PSQI)	Pre-lockdown	638	2.07 + 0.7	3.235	**0.001**
	During-lockdown		1.94 + 0.75		
Sleep latency (PSQI)	Pre-lockdown	636	2.3 + 0.96	−0.23	0.818
	During-lockdown		2.31 + 0.92		
Sleep duration (PSQI)	Pre-lockdown	637	2.08 + 1.05	6.648	**<0.001**
	During-lockdown		1.71 + 1.06		
Sleep efficiency (PSQI)	Pre-lockdown	638	2.15 + 1.1	0.473	0.636
	During-lockdown		2.12 + 1.05		
Sleep disturbance (PSQI)	Pre-lockdown	638	2.06 + 0.6	0.154	0.878
	During-lockdown		2.05 + 0.58		
Daytime dysfunction (PSQI)	Pre-lockdown	638	2.07 + 0.79	5.472	**<0.001**
	During-lockdown		1.85 + 0.79		
Sleep medication (PSQI)	Pre-lockdown	638	1.11 + 0.53	−1.507	0.132
	During-lockdown		1.22 + 0.53		
Anxiety (HADS-A)	Pre-lockdown	636	9.98 + 4.69	0.368	0.713
	During-lockdown		9.89 + 4.76		
Depression (HADS-D)	Pre-lockdown	636	9.09 + 4.40	3.233	**0.001**
	During-lockdown		8.35 + 4.44		
Pain interference (BPI)	Pre-lockdown	638	6.36 + 2.19	3.552	**<0.001**
	During-lockdown		5.96 + 2.32		
Pain severity (BPI)	Pre-lockdown	638	5.43 + 1.8	2.879	**0.004**
	During-lockdown		5.16 + 1.86		

SD: standard deviation; PSQI: Pittsburgh sleep quality index; HADS-A: Hospital Anxiety and Depression Scale (Anxiety); HADS-D: Hospital Anxiety and Depression Scale (Depression); BPI: brief pain inventory.

Statistically significant differences are in bold.

### Addressing Aim 2: Differences in wellbeing outcomes at Time 2

Three MANOVA were conducted using cross-sectional Time 2 data to determine whether there were any differences in wellbeing outcomes (sleep, anxiety and depression) based on changes in dependence on others, ability to self-manage pain, and access to healthcare, during the pandemic.

### Differences in wellbeing outcomes based on changes of dependence on others during the pandemic

The results of the first MANOVA presented in Table 3 yielded a significant main effect of dependence on others during the pandemic on global sleep quality, anxiety and depression; Pillai’s Trace = 0.02, *F*(6,1258) = 2.47, *p* = 0.02, *n*^2^ = 0.01. Between-subject effects showed significant group differences for sleep, anxiety and depression. Scheffe’s pairwise comparisons revealed that individuals whose dependence on others had increased during the pandemic reported significantly poorer sleep (*M* = 13.50) than individuals who reported less dependence on others during the pandemic (*M* = 12.60). A similar trend was observed with regards to anxiety scores. Individuals with increased dependence on others reported significantly higher anxiety scores (*M* = 10.37) than those who had become less dependent during the pandemic (*M* = 9.01). Finally, those who reported increased dependence on others, had significantly higher depression scores (*M* = 8.75) than those individuals who reported less dependence on others (*M* = 7.67).

**Table 3.** Between subject differences in global sleep quality, anxiety and depression based on dependence on others during the pandemic.

Variable	Dependence on others during pandemic (*M*, SD)					
	Increased *n* = 396	No change *n* = 92	Decreased *n* = 145	*F* (2, 630)	*p* Value	*n* ^2^
Sleep	13.50 (3.62)^a^	12.73 (3.89)^ab^	12.60 (3.90)^b^	3.89	0.021	0.01
Anxiety	10.37 (4.73)^a^	9.21 (4.42)^ab^	9.01 (4.90)^b^	5.57	0.004	0.02
Depression	8.75 (4.41)^a^	7.68 (4.38)^ab^	7.67 (4.43)^b^	4.40	0.013	0.01

*M*: mean score; SD: standard deviation.

Superscript letters have been used to indicate significant differences between-group Scheffe-corrected significance. Where letters are the same across variables there is no difference, and where letters differ (i.e. a and b), this denotes a significant difference (*p* ≤ 0.05). Participants with missing values for independent or dependent variables were excluded from this analysis.

### Differences in wellbeing outcomes based on ability to self-manage pain during the pandemic

The results of the second MANOVA presented in Table 4 yielded a significant main effect of ability to manage pain during the pandemic on global sleep quality, anxiety and depression; Pillai’s Trace = 0.08, *F*(6,1258) = 8.40, *p* =< 0.001, *n*^2^ = 0.04. Between-subject results yielded significant group differences for sleep, anxiety and depression. Scheffe’s pairwise comparisons revealed that individuals who found it harder to self-manage their pain during the pandemic had significantly poorer sleep (*M* = 13.79) than both individuals who reported no change (*M* = 12.39) and those who reported easier self-management of their pain during the pandemic (*M* = 10.92). Similarly, individuals who found it harder to self-manage their pain during the pandemic reported significantly higher levels of anxiety (*M* = 10.70) than both those who reported no change (*M* = 8.62) and those found it easier to self-manage their pain (*M* = 8.05). The same trend was observed in relation to difference in depression scores. Those who struggled more with pain-management reported significantly higher levels of depression (*M* = 8.89) than both those reporting no change (*M* = 7.55) and those finding pain-management easier during the pandemic (*M* = 6.78).

**Table 4.** Between subject differences in sleep quality, anxiety and depression based on ability to self-manage pain during pandemic.

Variable	Ability to manage pain during pandemic (*M*, SD)					
	Harder *n* = 397	No change *n* = 199	Easier *n* = 37	*F* (2, 630)	*p* Value	*n* ^2^
Sleep	13.79 (3.74)^a^	12.39 (3.89)^b^	10.92 (3.90)^b^	17.35	<0.001	0.05
Anxiety	10.70 (4.65)^a^	8.62 (4.76)^b^	8.05 (3.99)^b^	16.37	<0.001	0.05
Depression	8.89 (4.26)^a^	7.55 (4.77)^b^	6.78 (3.43)^b^	8.76	<0.001	0.03

*M*: mean score; SD: standard deviation.

Superscript letters have been used to indicate significant differences between-group Scheffe-corrected significance. Where letters are the same across variables there is no difference, and where letters differ (i.e. a and b), this denotes a significant difference (*p* ≤ 0.05). Participants with missing values for independent or dependent variables were excluded from this analysis.

The results of the third MANOVA presented in Table 5 yielded a significant main effect of access to healthcare during the pandemic (unrelated to COVID-19) on global sleep quality, anxiety and depression; Pillai’s Trace = 0.04, *F*(6,1190) = 4.12, *p* =< 0.001, *n*^2^ = 0.02. There were significant between subject differences for sleep, anxiety and depression. Scheffe’s pairwise comparisons revealed that individuals who had healthcare appointments cancelled during the pandemic reported significantly poorer sleep (*M* = 13.50) than those who had usual access to care (*M* = 12.05). The same trend was observed with regards to anxiety levels. Individuals with cancelled healthcare appointments reported higher levels of anxiety (*M* = 10.30) than those with usual access to care (*M* = 8.83). Finally, participants with usual access to care during the pandemic had significantly lower levels of depressive symptoms (*M* = 6.41) than both those who experienced cancelled healthcare appointments (*M* = 8.79) and those who had face to face appointments replaced with telephone or virtual appointments (*M* = 8.32).

**Table 5.** Between subject differences in sleep quality, anxiety and depression based on access to healthcare during the pandemic.

Variable	Access to healthcare during the pandemic (*M*, SD)					
	HC appointments cancelled *n* = 375	HC appointments remote/online *n* = 138	No Changes/no appointments *n = 86*	*F* (2, 596)	*p* Value	*n* ^2^
Sleep	13.50 (3.54)^a^	12.94 (3.92)^ab^	12.05 (4.15)^b^	5.71	0.003	0.02
Anxiety	10.30 (4.75)^a^	9.78 (4.79)^ab^	8.83 (4.60)^b^	3.47	0.032	0.01
Depression	8.79 (4.47)^a^	8.32 (4.08)^a^	6.41 (4.10)^b^	10.62	<0.001	0.03

HC: healthcare; M: mean score; SD: standard deviation.

Superscript letters have been used to indicate significant differences between-group Scheffe-corrected significance. Where letters are the same across variables there is no difference, and where letters differ (i.e. a and b), this denotes a significant difference (*p* ≤ 0.05). Participants with missing values for independent or dependent variables were excluded from this analysis.

## Discussion

Our prospective study describes the impact on wellbeing outcomes in a sample of 638 adults with chronic pain in the United Kingdom during the first wave of the COVID-19 pandemic. Despite increasing COVID-19 cases and related deaths between Time 1 and Time 2, statistically significant improvements in global sleep quality, subjective sleep quality, sleep duration, daytime dysfunction, depressive symptoms, pain interference, and pain severity were reported. At initial glance, it may seem these results contradict recent research findings which have reported an increase in sleep problems and mental health distress across general and clinical populations during the COVID-19 pandemic (Pierce et al., 2020; Wang et al., 2020b). However, our findings may reflect the difference in methodology used in other studies. Our study collected baseline data in the immediate period before government mandated lockdown measures were implemented and whilst the trend in cases was increasing exponentially. Follow-up measures were collected during lockdown, past the peak of case rises from COVID-19. One study from China conducted by Wang et al. (2020a) collected baseline and follow-up survey data during similar time points and found statistically significant reductions in PTSD symptoms between Time 1 and Time 2, as well as no changes in depression or anxiety scores. Although the Wang et al. (2020a) study was conducted on a general adult population, the methodology is comparable to the current study with regards to how and when data were collected during the outbreak in each respective country. The small improvements in sleep, depression, and pain outcomes between Time 1 and Time 2 may reflect feelings of uncertainty and health-related fear at the start of the outbreak, whilst there were upward trends in COVID-19 cases and deaths. The implementation of a nationwide lockdown and a downward trend in cases after mid-April 2020 may have contributed to some sense of security among our pain population thus resulting in the observed temporal changes in sleep, depression and pain scores. It is also possible that pain scores improved due to decrease in work related activities (e.g. furlough) and increased home-working practices.

Despite these improvements, scores for global sleep quality, anxiety and depression were above clinical cut-off points at Time 2 (Backhaus et al., 2002; Olssøn et al., 2005). Research has shown that individuals living with chronic pain are at higher risk for concomitant sleep problems, anxiety, and depression, with higher scores compared to the general population (Hooten, 2016; Smith and Haythornthwaite, 2004). Our results demonstrate that individuals who felt less dependent on others had fewer sleep problems, anxiety, and depressive symptoms compared to those who reported feeling more dependent on others for practical and emotional support during this time. Furthermore, those who reported difficulty managing their pain condition had higher levels of sleep problems, anxiety and depression than those who managed their condition with ease. Interestingly, the disparity between these two groups crossed the clinical threshold for depression scores; individuals who found it easier to self-manage their pain were, on average, below threshold for depressive symptoms (scores <8) and those who reported more difficulty in their pain self-management were above the clinical threshold (scores >8) (Zigmond and Snaith, 1994). Finally, regarding access to healthcare, individuals whose healthcare appointments had been cancelled reported higher levels of sleep problems, anxiety, and depression than those who had experienced no disruption to usual care. Once again for depression scores, the difference between those individuals whose appointments had been cancelled and those who had usual care crossed the clinical threshold (Zigmond and Snaith, 1994).

The above findings support previous studies which illustrate how problems in sleep, increased anxiety and depression may develop as a long-term sequalae of chronic pain. The results also demonstrate the disruption to independence, self-management and access to healthcare provision during the pandemic among this population. Chronic pain poses a threat to individuals’ perceived independence, and research has highlighted the importance of retaining independence in order to carry out activities of daily living and social interactions (Robinson et al., 2013; Tollefson et al., 2011). The ability to self-manage for individuals with chronic pain has been shown to reduce psychological distress and disability (Nicholas et al., 2012). Finally, our findings also outline the importance of maintaining access to health and care services as part of the wider care management plan for many with chronic pain.

### Implications and recommendations

Based on the findings discussed, we highlight implications related to psychosocial wellbeing, workforce, and healthcare practice along with practical recommendations which should be considered in the current climate. Firstly, it is clear that the lockdown measures implemented during the first wave of COVID-19 have impacted communities and support for special populations. Our research highlights the importance of retained independence on mental health in chronic pain populations. It is foreseen that future lockdowns will cause a social disconnection and a threat of increased loneliness (Karos et al., 2020). Thus, we recommended that considerations are made for allowing social ‘bubbles’ and social cohesion to continue in support for chronic pain communities during future public health restrictions. Secondly, a significant proportion of the UK workforce is affected by chronic pain and disability across all sectors and skills bases who contribute greatly to our economy (Fayaz et al., 2016b). It is therefore vital to ensure this portion of the workforce can continue their contribution whilst managing the increased risk of disease severity as a result of COVID-19. Workplace managers (supported by policies to protect workers’ rights) should carry out necessary risk assessment and ensure that individuals most at risk can contribute via adaptable working plans, whether through remote working or redeployment, before taking steps to prevent individuals from working in any capacity. Thirdly, there is evidence that closure of non-urgent health services has impacted waitlists as they begin to reopen (Karos et al., 2020). We therefore recommend that services which begin to triage backlogs of cases based on clinical need ensure referral pathways to psychological and mental health services are in place. There is evidence that mental health has suffered as a result of halted health and social care services and is it reasonable to assume that once financial assistance programmes come to an end (such as furlough schemes), many more will be in need of these services (Witteveen, 2020).

### Limitations

This research relied on self-report measures which are known to misalign with more objective measures and reporting. Despite this, self-report measures offer a quick and accessible solution to data gathering via remote means which was imperative during the pandemic. Secondly, our sample consisted of individuals with chronic pain conditions, and therefore it is not possible to relate our findings to the general population. However, it is hoped they will be relevant to informing ways to improve wellbeing for individuals with chronic pain during future periods of lockdown. Thirdly, the majority of participants identified as ‘White – any background’ ethnicity and is therefore limited in generalizability, future studies should seek to explore methods to engage more diversity in responses (Fryer et al., 2016). Fourthly, it was not possible to include SES in the analysis, future studies should consider SES as a control variable. Finally, our data were collected across two time points, only one of which occurred during the lockdown which resulted in some of our analyses being conducted on cross-sectional data. Future research should seek to collect data longitudinally throughout the pandemic to assess causal relationships between lockdown measures on wellbeing.

## Conclusion

To our knowledge, at time of writing, our study is the first to assess changes in wellbeing outcomes in a chronic pain population before and during the first lockdown in the UK. The results of this study suggest that there were small but significant improvements post-lockdown in relation to global sleep quality, depression and pain outcomes. Groups were identified as more likely to experience poorer wellbeing during lockdown based on increased dependence on others, lesser ability to self-manage pain, and restricted access to healthcare. As such, governing bodies and healthcare providers should enable essential services for chronic pain individuals as well as ensuring restrictions do not impact the ability for individuals to support themselves in managing their condition.

The research and learnings from the COVID-19 outbreak must be used to inform policy and emergency planning responses for future pandemics. Policymakers should consult with a wide range of professionals such as healthcare, social workers and Third Sector workers to plan local strategies which meet individual as well as collective needs within the population.

## Research Data

sj-docx-1-hpq-10.1177_1359105321995962 – Acute impact of a national lockdown during the COVID-19 pandemic on wellbeing outcomes among individuals with chronic pain Click here for additional data file. sj-docx-1-hpq-10.1177_1359105321995962 for Acute impact of a national lockdown during the COVID-19 pandemic on wellbeing outcomes among individuals with chronic pain by Zoë Zambelli, António R Fidalgo, Elizabeth J Halstead and Dagmara Dimitriou in Journal of Health Psychology This article is distributed under the terms of the Creative
Commons Attribution 4.0 License ( https://creativecommons.org/licenses/by/4.0/) which
permits any use, reproduction and distribution of the work without
further permission provided the original work is attributed as specified
on the SAGE and Open Access pages ( https://us.sagepub.com/en-us/nam/open-access-at-sage).

sj-sav-2-hpq-10.1177_1359105321995962 – Acute impact of a national lockdown during the COVID-19 pandemic on wellbeing outcomes among individuals with chronic pain Click here for additional data file. sj-sav-2-hpq-10.1177_1359105321995962 for Acute impact of a national lockdown during the COVID-19 pandemic on wellbeing outcomes among individuals with chronic pain by Zoë Zambelli, António R Fidalgo, Elizabeth J Halstead and Dagmara Dimitriou in Journal of Health Psychology This article is distributed under the terms of the Creative
Commons Attribution 4.0 License ( https://creativecommons.org/licenses/by/4.0/) which
permits any use, reproduction and distribution of the work without
further permission provided the original work is attributed as specified
on the SAGE and Open Access pages ( https://us.sagepub.com/en-us/nam/open-access-at-sage).

sj-spv-3-hpq-10.1177_1359105321995962 – Acute impact of a national lockdown during the COVID-19 pandemic on wellbeing outcomes among individuals with chronic pain Click here for additional data file. sj-spv-3-hpq-10.1177_1359105321995962 for Acute impact of a national lockdown during the COVID-19 pandemic on wellbeing outcomes among individuals with chronic pain by Zoë Zambelli, António R Fidalgo, Elizabeth J Halstead and Dagmara Dimitriou in Journal of Health Psychology This article is distributed under the terms of the Creative
Commons Attribution 4.0 License ( https://creativecommons.org/licenses/by/4.0/) which
permits any use, reproduction and distribution of the work without
further permission provided the original work is attributed as specified
on the SAGE and Open Access pages ( https://us.sagepub.com/en-us/nam/open-access-at-sage).

## References

[bibr1-1359105321995962] Arora T Grey I (2020) Health behaviour changes during COVID-19 and the potential consequences: A mini-review. Journal of Health Psychology 25(9): 1155–1163. 32551944 10.1177/1359105320937053

[bibr2-1359105321995962] Backhaus J Junghanns K Broocks A , et al (2002) Test–retest reliability and validity of the Pittsburgh Sleep Quality Index in primary insomnia. Journal of Psychosomatic Research 53(3): 737–740. 12217446 10.1016/s0022-3999(02)00330-6

[bibr3-1359105321995962] Bevers K Watts L Kishino ND , et al (2016) The biopsychosocial model of the assessment, prevention, and treatment of chronic pain. US Neurol 12(2): 98–104.

[bibr4-1359105321995962] Bjelland I Dahl AA Haug TT , et al (2002) The validity of the Hospital Anxiety and Depression Scale: An updated literature review. Journal of Psychosomatic Research 52(2): 69–77. 11832252 10.1016/s0022-3999(01)00296-3

[bibr5-1359105321995962] Buysse D (1989) The Pittsburgh Sleep Quality Index (PSQI): A New Instrument for Psychiatric Practice and Research. Pittsburgh: Elsevier Scientific Publishers Ireland. 10.1016/0165-1781(89)90047-4 2748771

[bibr6-1359105321995962] Carpenter JS Andrykowski MA (1998) Psychometric evaluation of the Pittsburgh sleep quality index. Journal of Psychosomatic Research 45(1): 5–13. 9720850 10.1016/s0022-3999(97)00298-5

[bibr7-1359105321995962] Chakraborty I Maity P (2020) COVID-19 outbreak: Migration, effects on society, global environment and prevention. Science of the Total Environment 728: 138882. 10.1016/j.scitotenv.2020.138882 PMC7175860 32335410

[bibr8-1359105321995962] Cleeland CS Ryan KM (1994) Pain assessment: Global use of the Brief Pain Inventory. Annals of the Academy of Medicine, Singapore 23(2): 129–138. 8080219

[bibr9-1359105321995962] Fayaz A Ayis S Panesar SS , et al (2016a) Assessing the relationship between chronic pain and cardiovascular disease: A systematic review and meta-analysis. Scandinavian Journal of Pain 13: 76–90. 28850537 10.1016/j.sjpain.2016.06.005

[bibr10-1359105321995962] Fayaz A Croft P Langford R , et al (2016b) Prevalence of chronic pain in the UK: A systematic review and meta-analysis of population studies. BMJ Open 6(6): e010364. 10.1136/bmjopen-2015-010364 PMC4932255 27324708

[bibr11-1359105321995962] Fryer CS Passmore SR Maietta RC , et al (2016) The symbolic value and limitations of racial concordance in minority research engagement. Qualitative Health Research 26(6): 830–841. 25769299 10.1177/1049732315575708 PMC4658313

[bibr12-1359105321995962] Gatchel RJ (2004) Comorbidity of chronic pain and mental health disorders: The biopsychosocial perspective. American Psychologist 59(8): 795. 10.1037/0003-066X.59.8.795 15554853

[bibr13-1359105321995962] Giordani RCF Zanoni da Silva M Muhl C , et al (2020) Fear of COVID-19 scale: Assessing fear of the coronavirus pandemic in Brazil. Journal of Health Psychology. Epub ahead of print 16 December 2020. DOI: 10.1177/1359105320982035. 33327789

[bibr14-1359105321995962] Gupta R Grover S Basu A , et al (2020) Changes in sleep pattern and sleep quality during COVID-19 lockdown. Indian Journal of Psychiatry 62(4): 370. 33165382 10.4103/psychiatry.IndianJPsychiatry_523_20 PMC7597722

[bibr15-1359105321995962] Hardman R Begg S Spelten E (2020) What impact do chronic disease self-management support interventions have on health inequity gaps related to socioeconomic status: A systematic review. BMC Health Services Research 20(1): 1–15. 10.1186/s12913-020-5010-4 PMC7045733 32106889

[bibr16-1359105321995962] Hooten WM (2016) Chronic pain and mental health disorders: Shared neural mechanisms, epidemiology, and treatment. Mayo Clinic Proceedings 91(7): 955–970. 27344405 10.1016/j.mayocp.2016.04.029

[bibr17-1359105321995962] Iacobucci G (2020) Covid-19: All non-urgent elective surgery is suspended for at least three months in England. BMJ: British Medical Journal 368: m1106. 32188602 10.1136/bmj.m1106

[bibr18-1359105321995962] Jarvis CI Van Zandvoort K Gimma A , et al (2020) Quantifying the impact of physical distance measures on the transmission of COVID-19 in the UK. BMC Medicine 18: 1–10. 32375776 10.1186/s12916-020-01597-8 PMC7202922

[bibr19-1359105321995962] Kang C Yang S Yuan J , et al (2020) Patients with chronic illness urgently need integrated physical and psychological care during the COVID-19 outbreak. Asian Journal of Psychiatry 51: 102081. 32289729 10.1016/j.ajp.2020.102081 PMC7139251

[bibr20-1359105321995962] Karos K McParland JL Bunzli S , et al (2020) The social threats of COVID-19 for people with chronic pain. Pain 161(10): 2229–2235. 32694381 10.1097/j.pain.0000000000002004 PMC7382418

[bibr21-1359105321995962] Liang L Gao T Ren H , et al (2020) Post-traumatic stress disorder and psychological distress in Chinese youths following the COVID-19 emergency. Journal of Health Psychology 25(9): 1164–1175. 32627606 10.1177/1359105320937057 PMC7342938

[bibr22-1359105321995962] Lu T Li Y Pan J , et al (2013) Study on minimal important difference of the Pittsburgh sleep quality index based on clinical trial of traditional Chinese medicine. Journal of Guangzhou University of Traditional Chinese Medicine: 4.

[bibr23-1359105321995962] Mease PJ Spaeth M Clauw DJ , et al (2011) Estimation of minimum clinically important difference for pain in fibromyalgia. Arthritis Care & Research 63(6): 821–826. 21312349 10.1002/acr.20449

[bibr24-1359105321995962] Morin CM Gibson D Wade J (1998) Self-reported sleep and mood disturbance in chronic pain patients. The Clinical Journal of Pain 14(4): 311–314. 9874009 10.1097/00002508-199812000-00007

[bibr25-1359105321995962] Nicholas MK Asghari A Corbett M , et al (2012) Is adherence to pain self-management strategies associated with improved pain, depression and disability in those with disabling chronic pain? European Journal of Pain 16(1): 93–104. 21705246 10.1016/j.ejpain.2011.06.005

[bibr26-1359105321995962] Nicholas MK Blyth FM (2016) Are self-management strategies effective in chronic pain treatment? Pain Management 6(1): 75–88. 26678703 10.2217/pmt.15.57

[bibr27-1359105321995962] Odriozola-González P Planchuelo-Gómez Á Irurtia MJ , et al (2020) Psychological symptoms of the outbreak of the COVID-19 confinement in Spain. Journal of Health Psychology. Epub ahead of print 30 October 2020. DOI: 10.1177/1359105320967086. 33124471

[bibr28-1359105321995962] Olssøn I Mykletun A Dahl AA (2005) The hospital anxiety and depression rating scale: A cross-sectional study of psychometrics and case finding abilities in general practice. BMC Psychiatry 5(1): 46. 16351733 10.1186/1471-244X-5-46 PMC1343544

[bibr29-1359105321995962] Ornell F Schuch JB Sordi AO , et al (2020) “Pandemic fear” and COVID-19: Mental health burden and strategies. Brazilian Journal of Psychiatry 42(3): 232–235. 32267343 10.1590/1516-4446-2020-0008 PMC7236170

[bibr30-1359105321995962] Pierce M Hope H Ford T , et al (2020) Mental health before and during the COVID-19 pandemic: A longitudinal probability sample survey of the UK population. The Lancet Psychiatry 7(10): 883–892. 32707037 10.1016/S2215-0366(20)30308-4 PMC7373389

[bibr31-1359105321995962] Puhan MA Frey M Büchi S , et al (2008) The minimal important difference of the hospital anxiety and depression scale in patients with chronic obstructive pulmonary disease. Health and Quality of Life Outcomes 6(1): 46. 18597689 10.1186/1477-7525-6-46 PMC2459149

[bibr32-1359105321995962] Reid MC Eccleston C Pillemer K (2015) Management of chronic pain in older adults. BMJ 350: h532. 25680884 10.1136/bmj.h532 PMC4707527

[bibr33-1359105321995962] Rhodes A Martin S Guarna J , et al (2020) A contextual-behavioral perspective on chronic pain during the COVID-19 pandemic and future times of mandated physical distancing. Journal of Contextual Behavioral Science 17: 152–158.

[bibr34-1359105321995962] Robinson K Kennedy N Harmon D (2013) Constructing the experience of chronic pain through discourse. Scandinavian Journal of Occupational Therapy 20(2): 93–100. 23016628 10.3109/11038128.2012.720275

[bibr35-1359105321995962] Smith MT Haythornthwaite JA (2004) How do sleep disturbance and chronic pain inter-relate? Insights from the longitudinal and cognitive-behavioral clinical trials literature. Sleep Medicine Reviews 8(2): 119–132. 15033151 10.1016/S1087-0792(03)00044-3

[bibr36-1359105321995962] Tan G Jensen MP Thornby JI , et al (2004) Validation of the brief pain inventory for chronic nonmalignant pain. The Journal of Pain 5(2): 133–137. 15042521 10.1016/j.jpain.2003.12.005

[bibr37-1359105321995962] Tollefson J Usher K Foster K (2011) Relationships in pain: The experience of relationships to people living with chronic pain in rural areas. International Journal of Nursing Practice 17(5): 478–485. 21939479 10.1111/j.1440-172X.2011.01963.x

[bibr38-1359105321995962] Treede R-D Rief W Barke A , et al (2015) A classification of chronic pain for ICD-11. Pain 156(6): 1003. 10.1097/j.pain.0000000000000160 PMC4450869 25844555

[bibr39-1359105321995962] Wang C Pan R Wan X , et al (2020a) A longitudinal study on the mental health of general population during the COVID-19 epidemic in China. Brain, Behavior, and Immunity 87: 40–48. 10.1016/j.bbi.2020.04.028 PMC7153528 32298802

[bibr40-1359105321995962] Wang Y Duan Z Ma Z , et al (2020b) Epidemiology of mental health problems among patients with cancer during COVID-19 pandemic. Translational Psychiatry 10(1): 1–10. 32737292 10.1038/s41398-020-00950-y PMC7393344

[bibr41-1359105321995962] Witteveen D (2020) Sociodemographic inequality in exposure to COVID-19-induced economic hardship in the United Kingdom. Research in Social Stratification and Mobility 69: 100551. 32921869 10.1016/j.rssm.2020.100551 PMC7474909

[bibr42-1359105321995962] Xiong J Lipsitz O Nasri F , et al (2020) Impact of COVID-19 pandemic on mental health in the general population: A systematic review. Journal of Affective Disorders 277: 55–64. 32799105 10.1016/j.jad.2020.08.001 PMC7413844

[bibr43-1359105321995962] Zigmond A Snaith R (1994) The HADS: Hospital Anxiety and Depression Scale. Windsor: NFER Nelson.

[bibr44-1359105321995962] Zigmond AS Snaith RP (1983) The hospital anxiety and depression scale. Acta Psychiatrica Scandinavica 67(6): 361–370. 6880820 10.1111/j.1600-0447.1983.tb09716.x

