# Supplementary material for: Acute impact of a national lockdown during the COVID-19 pandemic on wellbeing outcomes among individuals with chronic pain
Source: J Health Psychol. 2021 Feb 18;27(5):1099–110. doi: 10.1177/1359105321995962 (PMC8978477; doi:10.1177/1359105321995962)

**MANOVA: Access to Healthcare**

GLM CV1_PSQI_GlobalScore CV1_HADS_Anxiety CV1_HADS_Depression BY AccessToHealthcare

/METHOD=SSTYPE(3)

/INTERCEPT=INCLUDE

/POSTHOC=AccessToHealthcare(SCHEFFE)

/PLOT=PROFILE(AccessToHealthcare) TYPE=LINE ERRORBAR=NO MEANREFERENCE=NO YAXIS=AUTO

/EMMEANS=TABLES(AccessToHealthcare)

/PRINT=DESCRIPTIVE ETASQ OPOWER

/CRITERIA=ALPHA(.05)

/DESIGN= AccessToHealthcare.


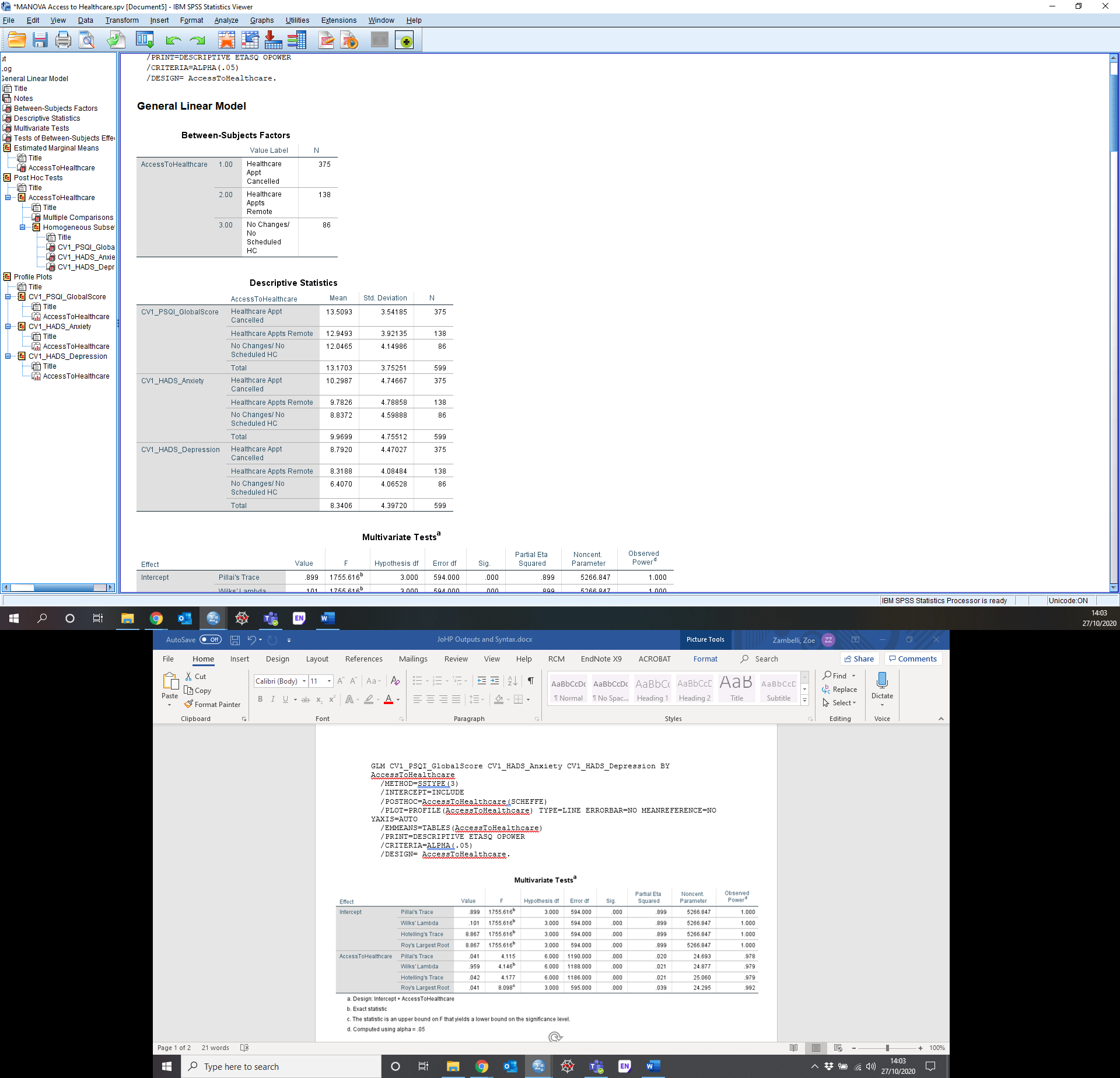


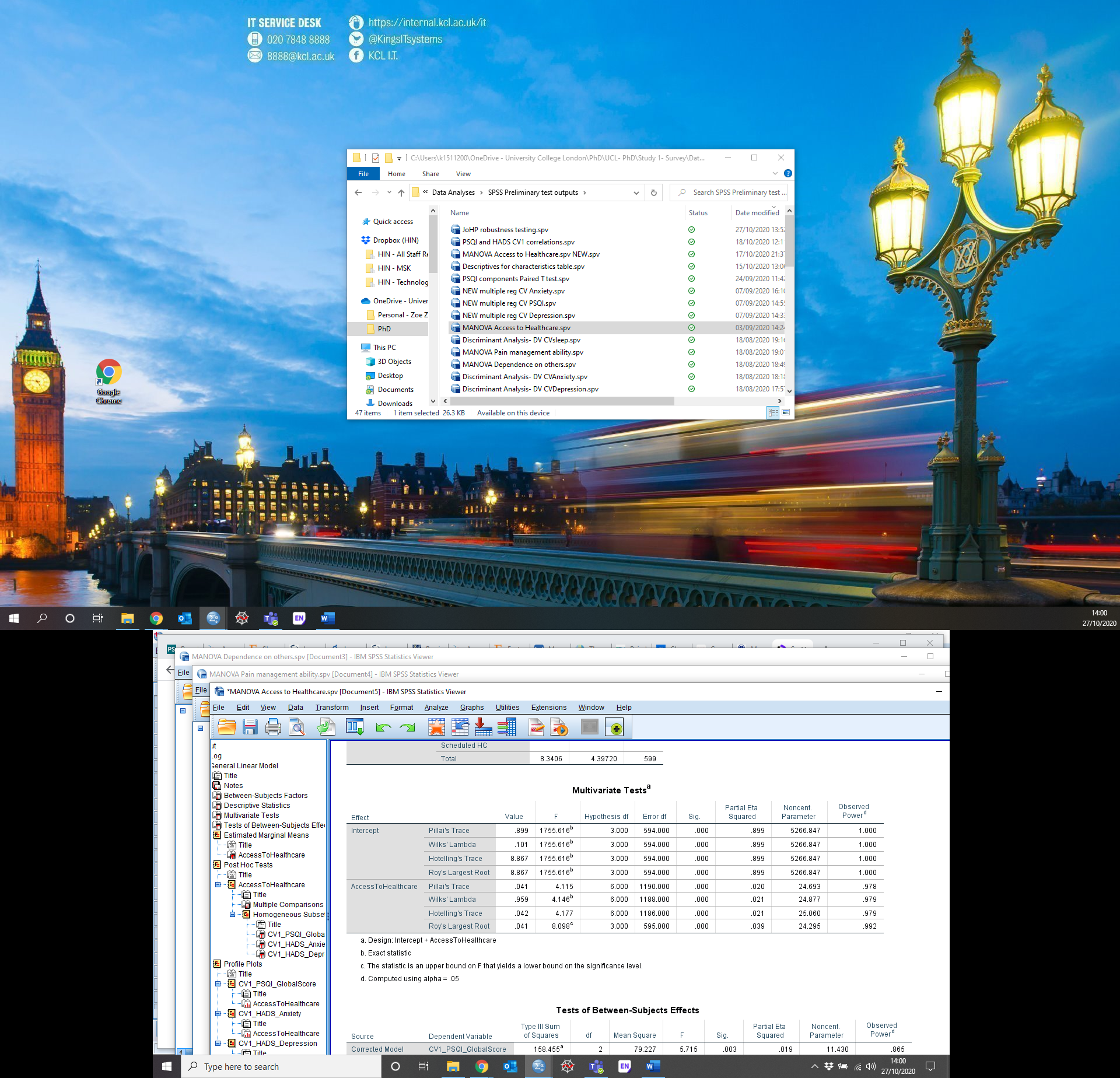


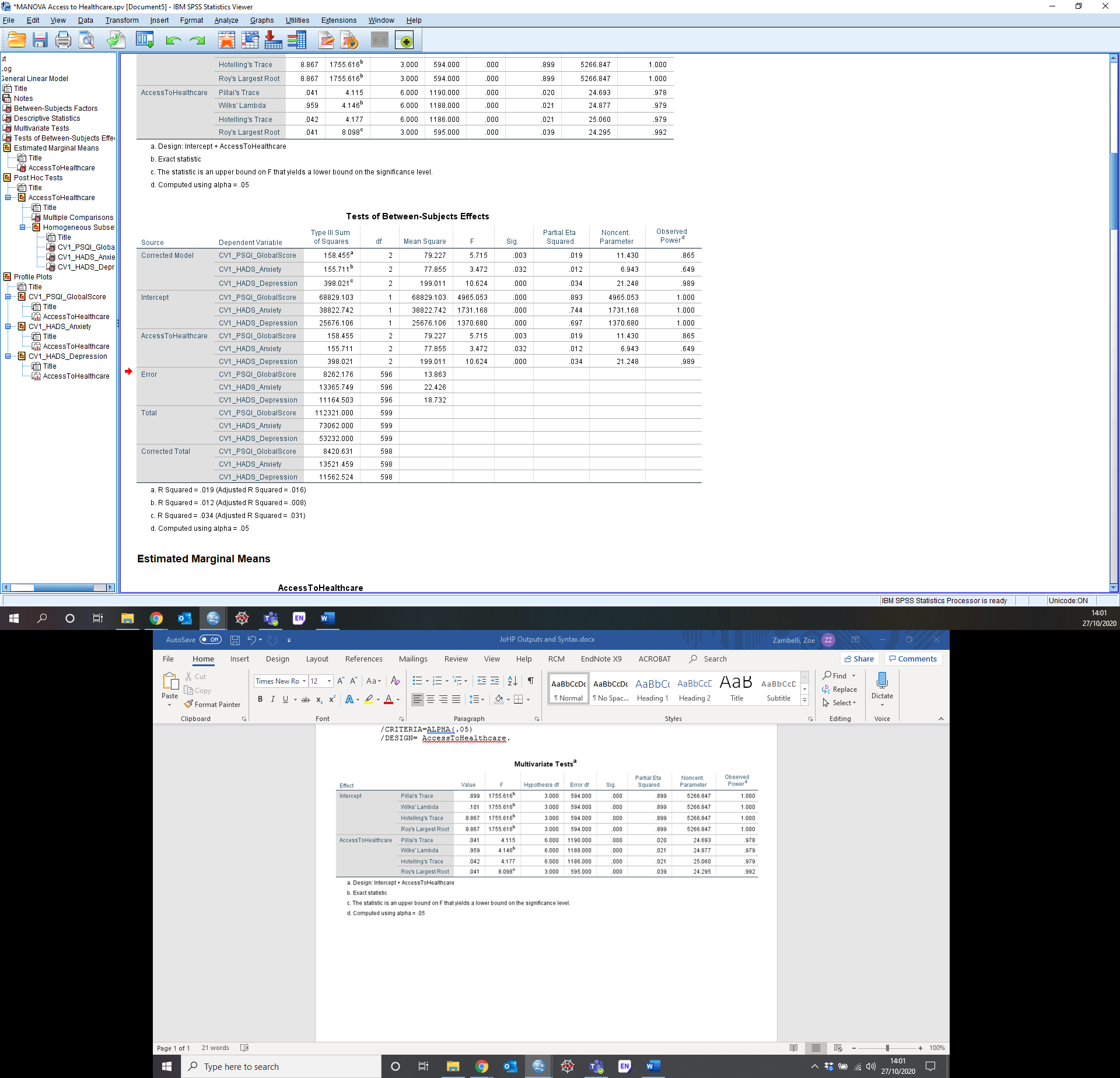


**MANOVA: Pain Management**

GLM CV1_HADS_Depression CV1_HADS_Anxiety CV1_PSQI_GlobalScore BY CV1_PainMngmtCats

/METHOD=SSTYPE(3)

/INTERCEPT=INCLUDE

/POSTHOC=CV1_PainMngmtCats(SCHEFFE)

/PRINT=DESCRIPTIVE ETASQ OPOWER HOMOGENEITY

/CRITERIA=ALPHA(.05)

/DESIGN= CV1_PainMngmtCats.


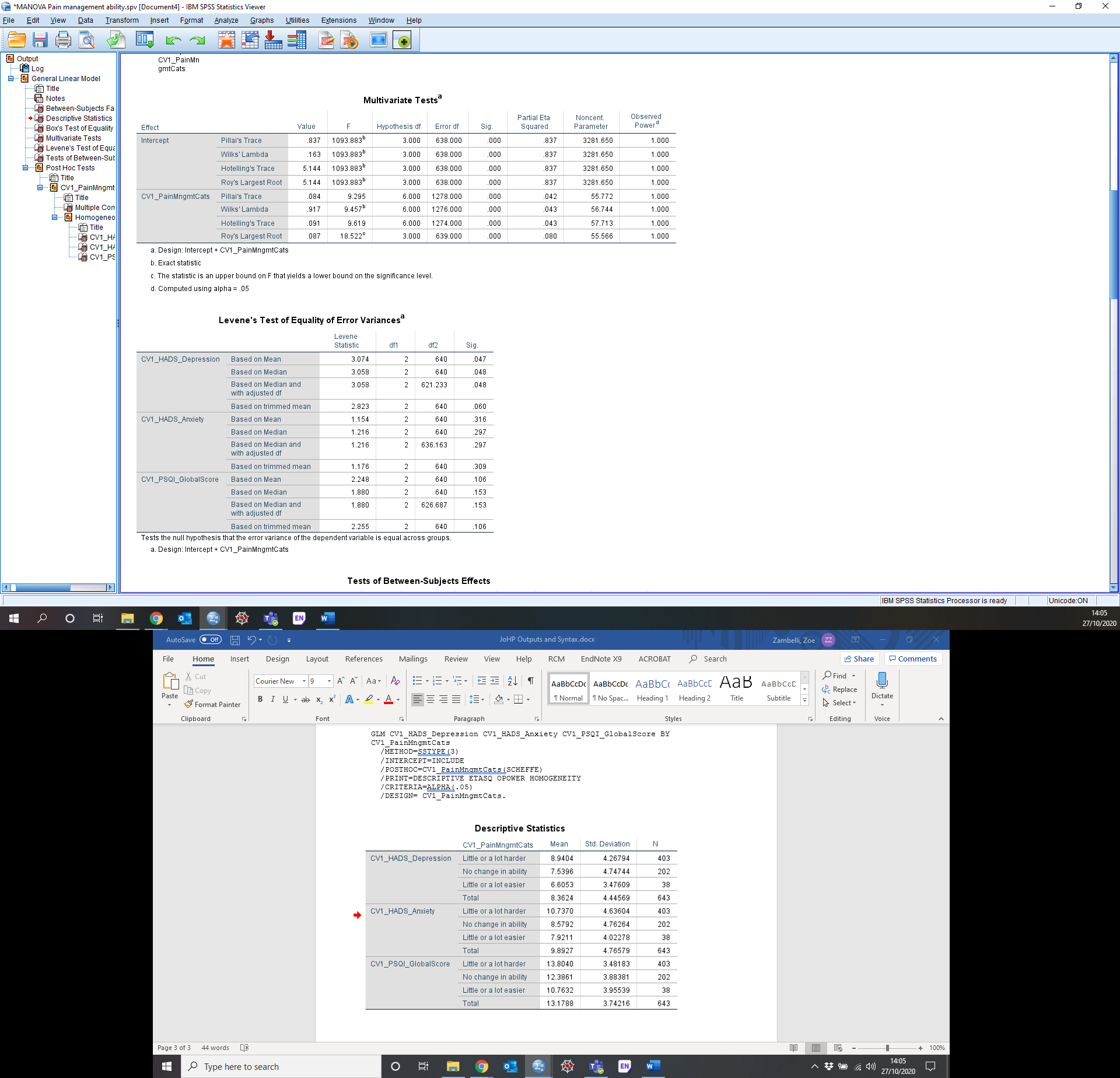

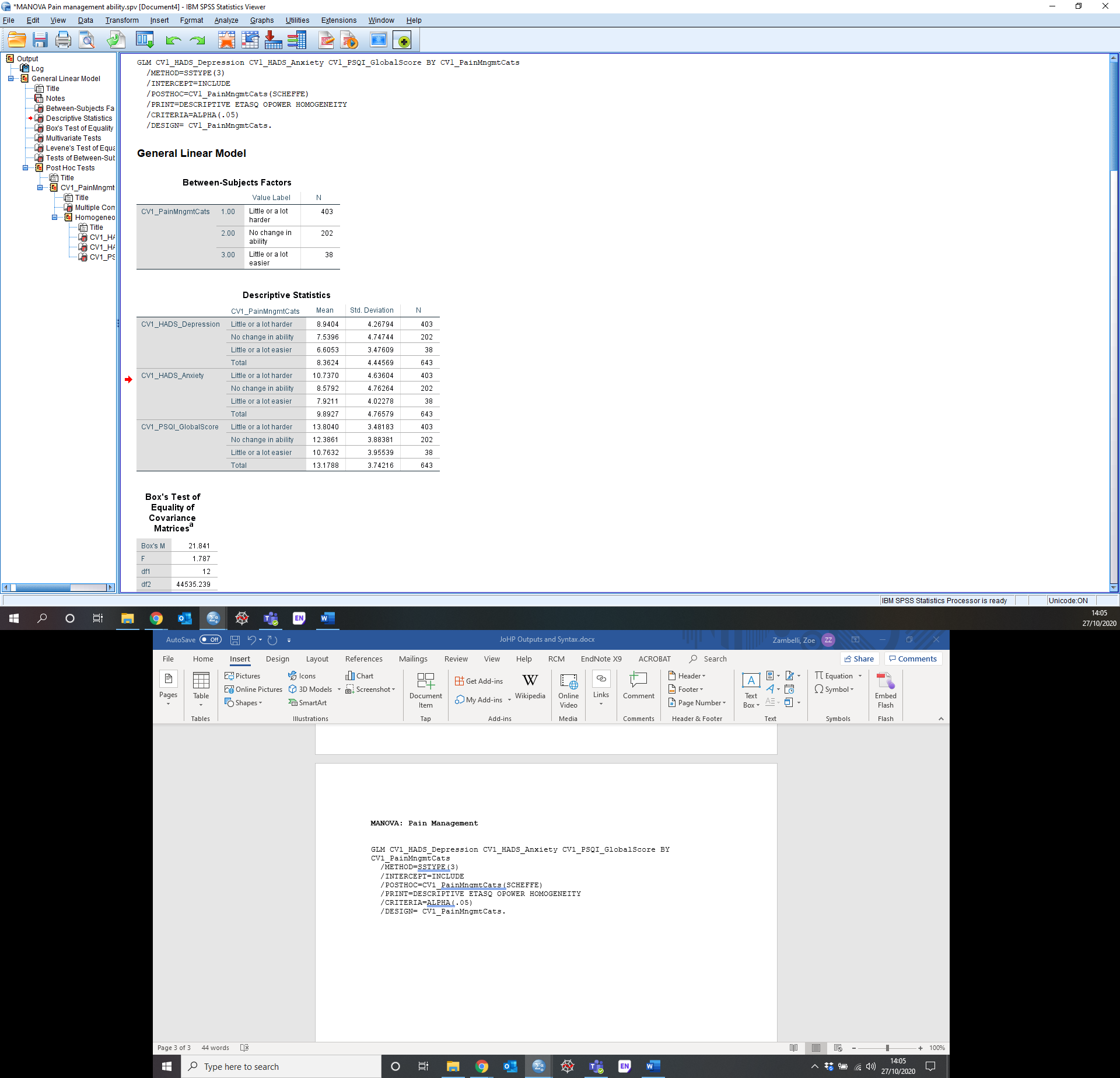


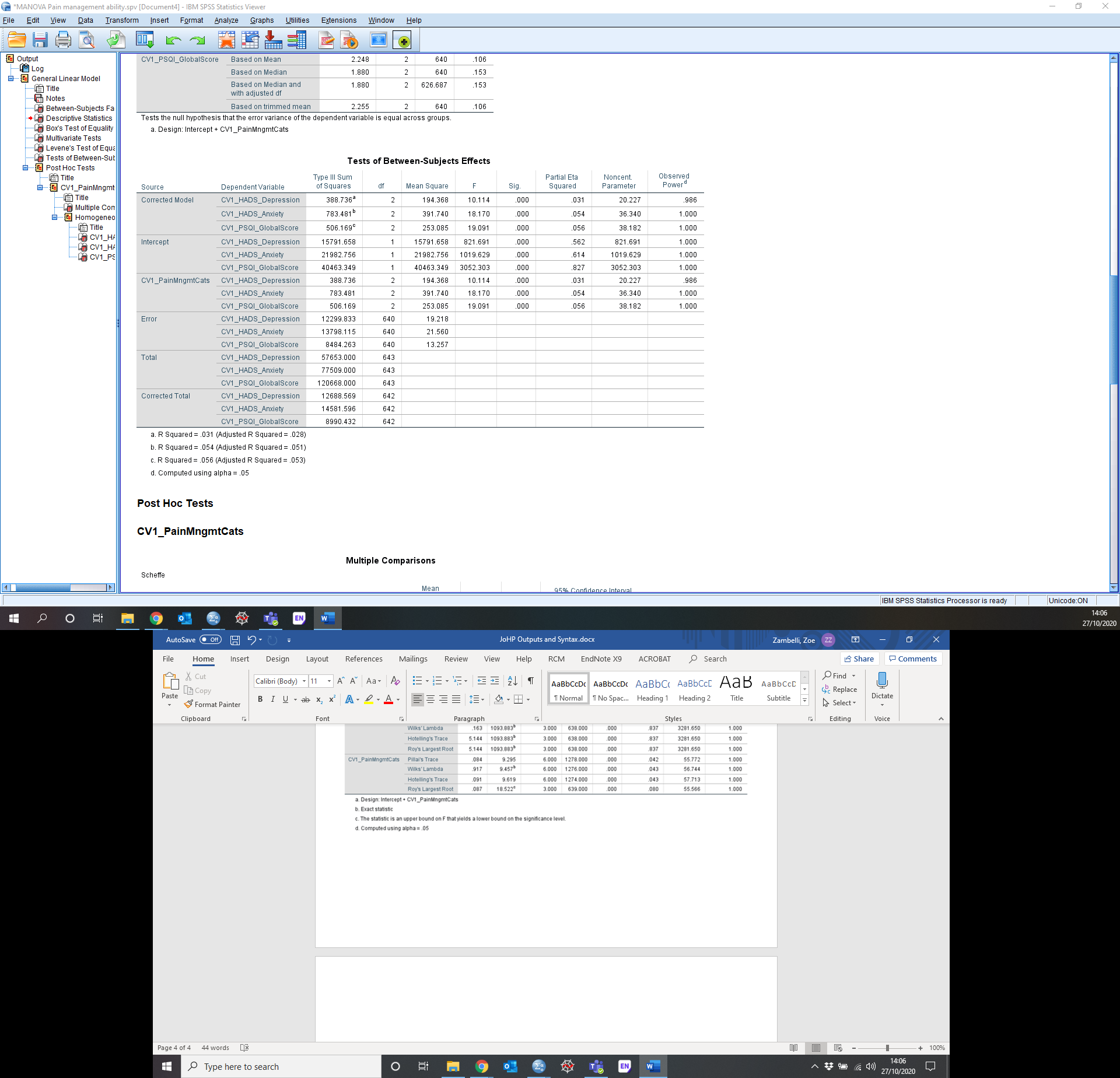


**MANOVA: Dependence on others**

GLM CV1_HADS_Depression CV1_HADS_Anxiety CV1_PSQI_GlobalScore BY CV1_DependenceOnOthers

/METHOD=SSTYPE(3)

/INTERCEPT=INCLUDE

/POSTHOC=CV1_DependenceOnOthers(SCHEFFE)

/PRINT=DESCRIPTIVE ETASQ OPOWER HOMOGENEITY

/CRITERIA=ALPHA(.05)

/DESIGN= CV1_DependenceOnOthers.


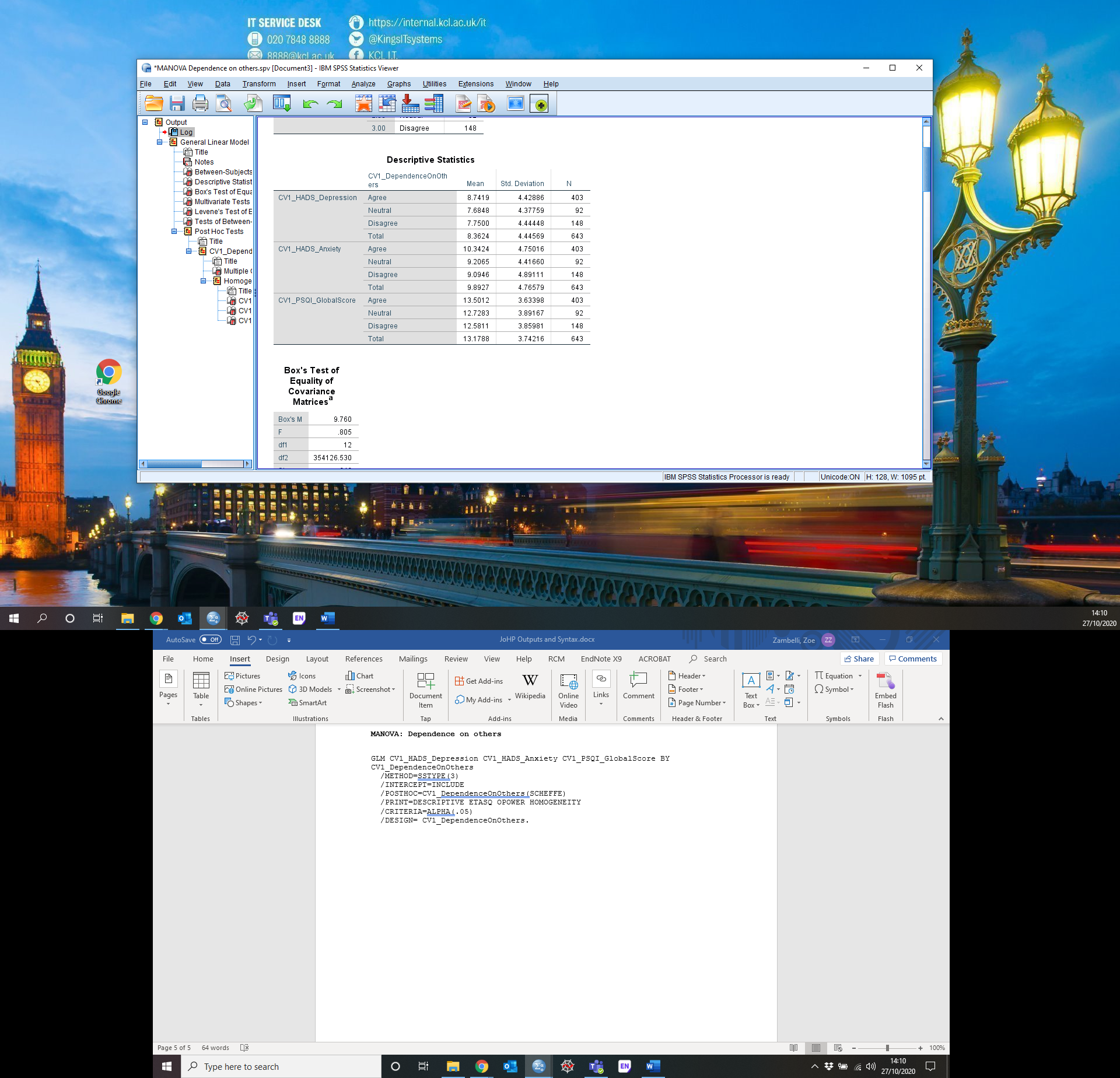


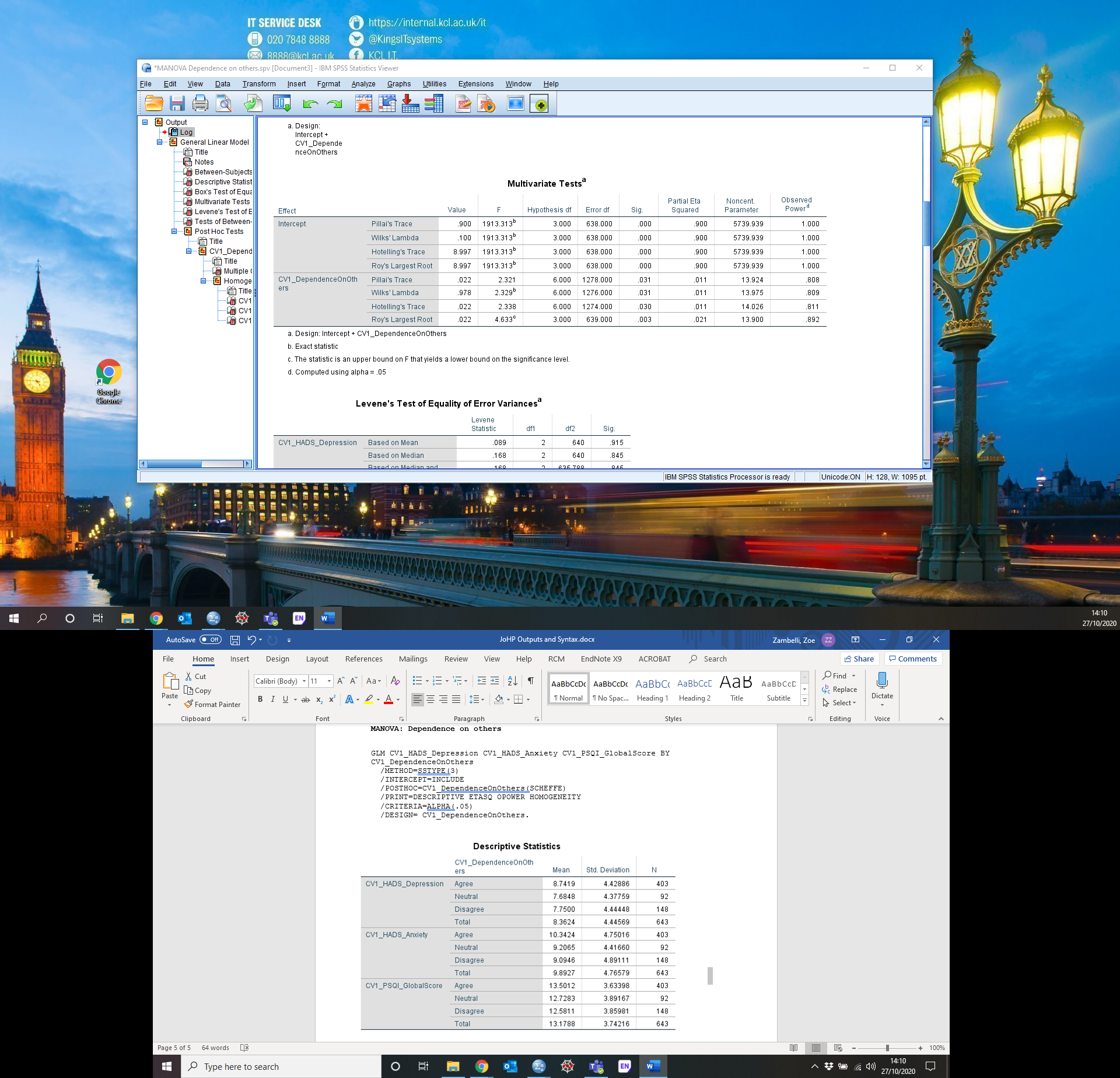


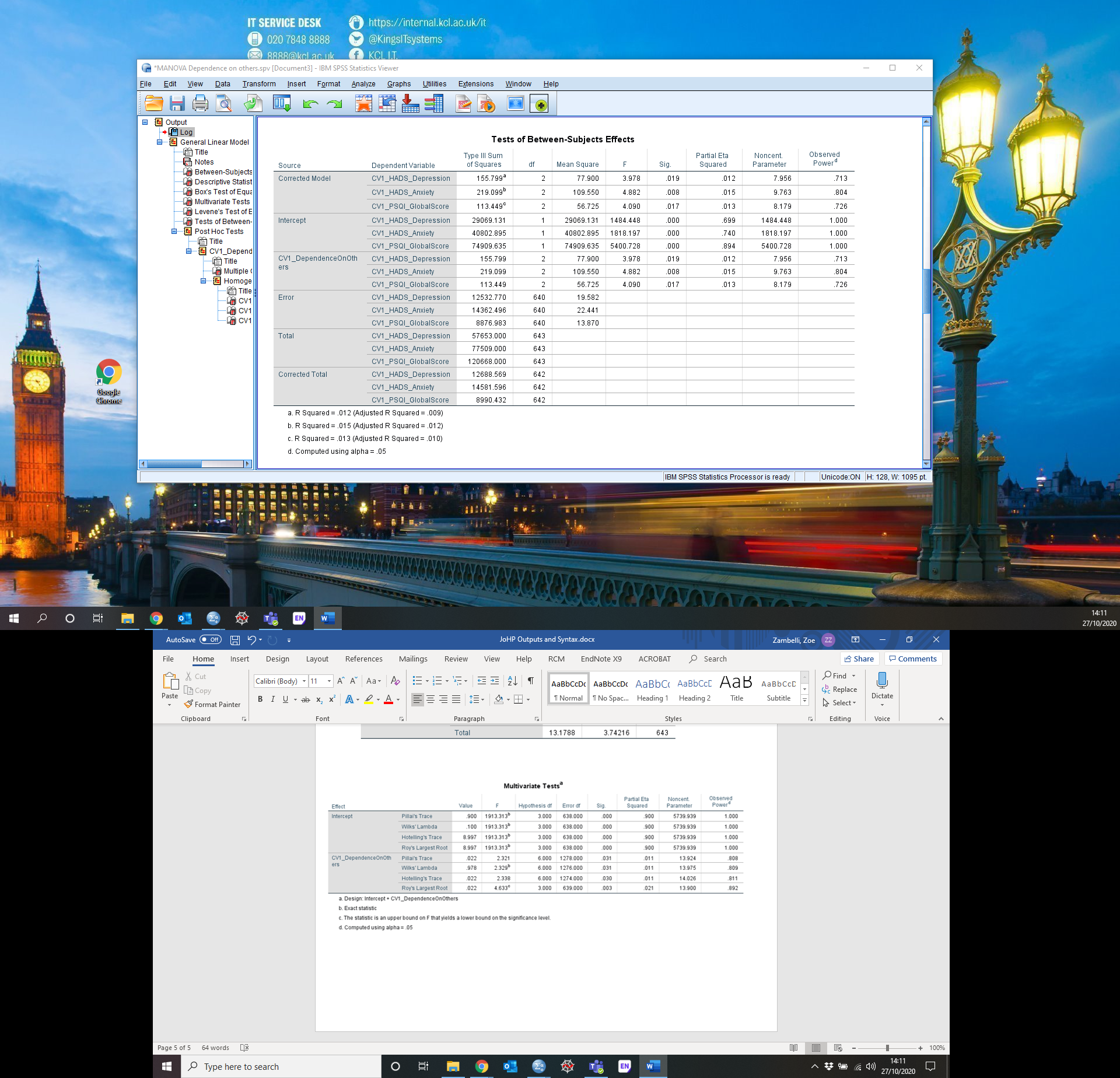

Supplement: sj-docx-1-hpq-10.1177_1359105321995962 – Acute impact of a national lockdown during the COVID-19 pandemic on wellbeing outcomes among individuals with chronic pain [file sj-docx-1-hpq-10.1177_1359105321995962.docx]
